# Perspectives on the progress of China’s 2009 – 2012 health system reform

**Published:** 2011-12

**Authors:** David Hipgrave

**Affiliations:** Nossal Institute for Global Health, University of Melbourne, Melbourne, Australia; * Formerly UNICEF China Chief of Health, Nutrition, and Water and Environmental Sanitation

In July this year, China’s Ministry of Health (MoH) commissioned an independent external mid-term review of progress in its current health system reform (HSR), itself designed after unprecedented input from six external agencies and the general public (1,2). In its draft report, as yet unreleased, the six-member team of eminent reviewers praises the leadership and financial commitment demonstrated by China’s government in pursuing HSR, but among other things makes strong calls for harmonisation of urban and rural insurance schemes, further improvements to payment mechanisms and oversight of the quality of care, quantitative evaluations of key systems outputs and health outcomes and scientific evaluation of the benefit and cost-effectiveness of traditional Chinese medicine.

The report will be complemented by a formal internal review of the HSR commissioned by China’s State Council. Previously such reports have comprised quantitative compilation of the funds allocated, facilities constructed, personnel trained and deployed, population insured and related benefits accruing, such as reduced service costs and household expenditure on health. This internal review will be informed by a “lite” version of China’s five-yearly National Health Services Survey (3), focusing less on the formal Survey (next due in 2013) on health status but more on operational, funding and implementation issues related to the HSR. However, as the external review and a perusal of recent published literature makes clear, there is currently little new research upon which to quantitatively assess specific outcomes, even those directly benefiting from HSR-funded programs such as cancer screening, aged care and cataract surgery, particularly in the absence of related denominators.

China’s HSR was a response to the deep inequity resulting from three decades of marketisation and de facto privatisation of the health sector (4), but despite massive injection of government funds and high uptake of insurance, out of pocket expenses for purchase of care remain witheringly high for the majority as costs rise (5), and the impact of pilot schemes and insurance is uncertain (6,7). A large reduction in the proportion of total health expenditure that is paid out-of-pocket, from a peak of 60% in 2001 to 36% in 2010 (Zhao Yuxin, China National Health Development Research Centre, personal communication), does not prove that population health needs are being met, only that government and insurance are funding a bigger proportion of services purchased. Although around a half of China’s population lives in rural areas, total health expenditure and government allocations heavily favour urban areas (8). In addition, the reforms have not tackled the health risks associated with smoking, environmental pollution, urbanisation and China’s aging population – which will collectively add hugely to total health expenditure (5,9), offsetting the benefits of reform.

Notwithstanding scant objective assessment of progress, China’s government is not only moving forward with HSR, but expanding its content, ambition and the resources allocated. China will shortly allocate additional finds to the 1.4 trillion renminbi (RMB) (around US$ 200 billion, € 150 billion) already provided (5), including new funds focusing on two of the five reform pillars targeting those most disadvantaged by existing inequity – primary health care delivery (10) and public health (11). It is no exaggeration to say that the government aims to establish, by 2020, a system of primary and preventive health care equivalent to that which evolved over many decades in developed nations.

Both these areas depend heavily on the front-line health workers formerly known as “barefoot doctors”, whose basic education and training are very low compared to qualified doctors or even public health nurses in most developed and many developing countries. Indeed, the poor quality of care and profit motive underlying most medical treatment by village doctors in China (4) partially underlies another of the five pillars, reform of the national essential medicines scheme (12). Recognising their key role, the government is increasingly raising expectations of the role village doctors will play. A recent pronouncement by the State Council assigns to them roles previously restricted to higher levels of the health system (10).

**Figure:**
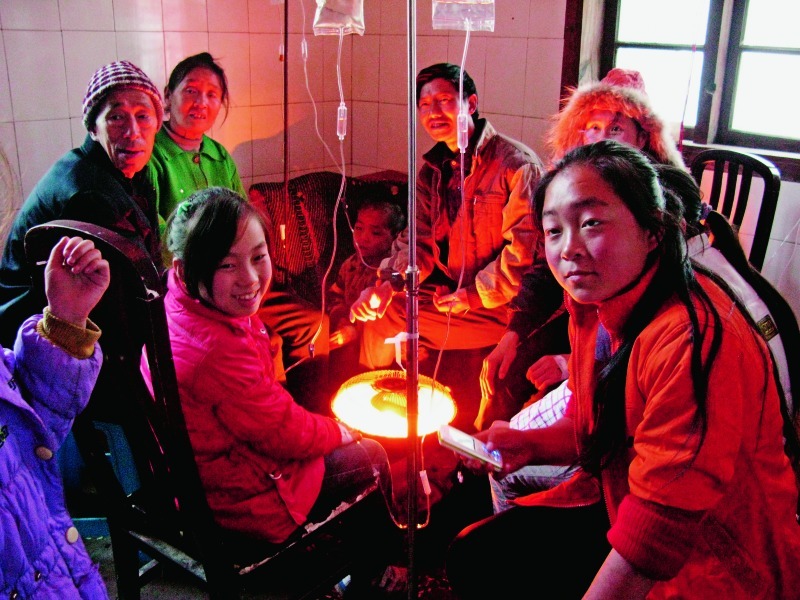
Photo: Courtesy of Dr David Hipgrave, personal collection

Under the new guidance, village doctors will be expected to provide progressively higher quality and standardised clinical care within the constraints of China’s new, zero profit approach to prescription of essential drugs (12). They will also undertake at least the ten basic public health activities (including aged-care, mental health, maternal and child health, vaccination and others) for which now RMB 25 (USD 3.9, € 2.9) per capita has been allocated across rural China, up from RMB 15 (USD 2.3, € 1.8) at the beginning of the HSR in April 2009 (11); participate in communicable disease surveillance and the national notifiable disease reporting system; administer payments through the cooperative medical (insurance) scheme (CMS) and participate in China’s new computerised health management and information system (HMIS) (13), using networked computers and new software. Their payment to implement this impressive list of activities will emanate from a combination of locally-funded compensation for lost drug income, standardised fees paid by the CMS for outpatient services by diagnostic group or capitation (piloting of which is in its earliest stages in China), and subsidies taken from the RMB25 per capita for implementation of public health services. Administration of these various income sources will be the responsibility of the county health bureau, also charged with ensuring village doctors’ qualification, licensing and continuing education (10).

These monumental changes will bring rural health care in China into the 21st century, as befits the nation’s status as a middle-income country and global economic giant. However, they connote expectations of diagnostic and therapeutic skill, public health competence, computer literacy, community participation and also administrative capacity at county level that the HSR external reviewers and people familiar with health care in rural China know are massively out of step with the status quo. In addition, given the State Council’s stated expectation that supervision of village doctors will be undertaken by township and county-level professional colleagues, and that their funding will be augmented by provincial and county resources (10), this modernisation of grassroots health care in China should be viewed as a generational process.

**Figure:**
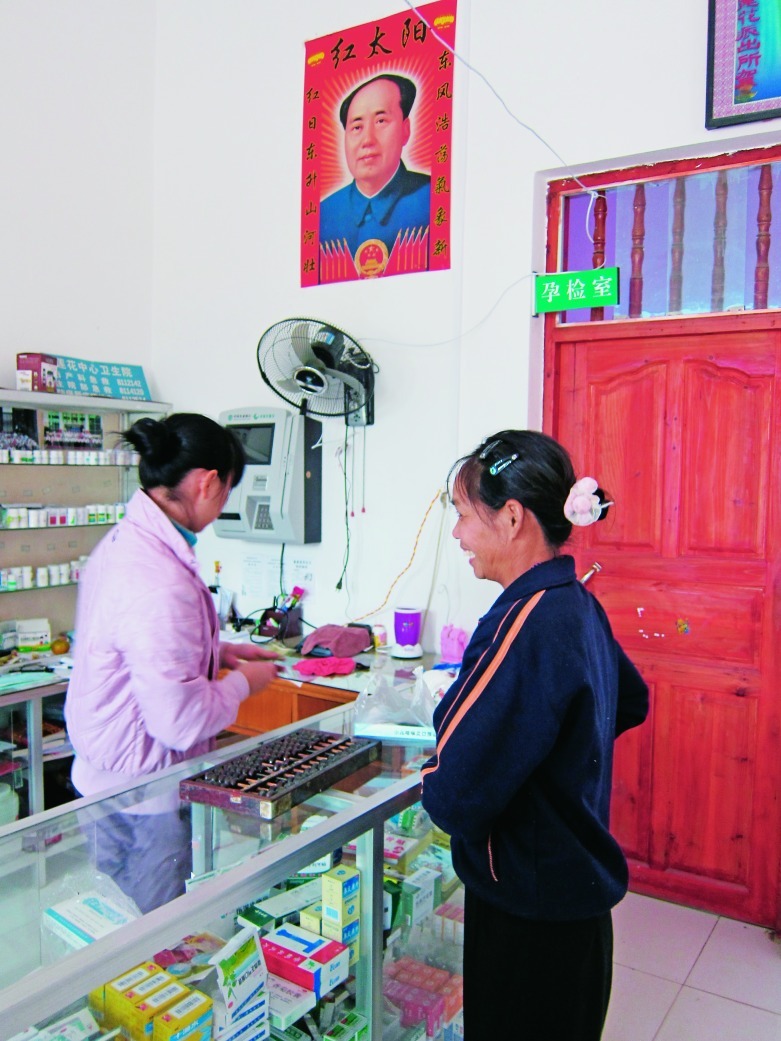
Photo: Courtesy of Kit Yee Chan, personal collection

Does that make it impossible? Only the brave would predict failure on anything so clearly supported at the highest level in modern China, but this brings attention to an old theme. In China’s system of policy centralisation with financial decentralisation (8), only the highest and most politicised national priorities have a strong likelihood of being implemented at the standard desired, and indeed local adaptation of national policy guidelines is encouraged in China (14,15). In the health sector, in recent years probably only the response to the SARS crisis was pursued with the level of priority finally allocated to it by the Chinese government. Most other health policies, recommendations, strategies or guidelines including the current HSR rely heavily on being prioritised and funded by local leaders, and this cannot be assumed. As in most developing nations, population health is simply not important enough to many local leaders in China, where economic development (ie the generation, not the consumption of income) is the main objective, especially in poorer areas (5). In addition, where private sector regulation is involved the situation becomes even more uncertain; for example oversight of food safety and the pharmaceutical industry are both very much prioritised by the Ministry of Health, but rely heavily on local authorities and the participation of other sectors and interests (16,17) [witness the ongoing concerns with the dairy industry almost three years after the original melamine-tainting outcry (18,19), and recent drug-, vaccine- and food-safety scandals (20-26)]. China’s HSR may be one of the highest profile and widely supported government initiatives of recent years, but the level of reliance on local funding and implementation and on other sectors suggests its success is not a given. Encouragingly, although substantive, independent assessment of the HSR and including the perspective of citizens is lacking, there are signs of increasing consultation and attention to public opinion in many areas of public policy in China (2,27-33), making it harder for local authorities to ignore national priorities. Moreover, China’s massive new online HMIS will record individualised data on health status, service uptake and payment, insurance participation and benefit, operational outputs and population-level health outcomes – all potentially in real-time (13). Failure of local support for HSR will thus become increasingly obvious as the HMIS is rolled out.

What then, of the possibility of an improved and more equitable health sector in China? In fact, like HSR in most nations, the outcome probably depends less on the MoH (which understands well the ends and is gradually devising the means to reach them), than on how other sectors and senior planners of China’s socio-economic trajectory perceive the importance of population health and equity. Whilst China must certainly play catch-up with developed nations in regard to educating its health workforce, standardising quality of care, improving data systems and devising a fair system to fund and pay for services, at least it has decades of experience from other nations to draw on in these areas. Payment for care is a particular area of discussion: whilst current initiatives are directing payment to providers like hospitals and village doctors, possibly augmenting perverse incentives to provide unnecessary care, such as Caesarean section (34), an opposing perspective would direct funding toward purchasers of care, whether individuals or insurers, to encourage efficiency and quality (5,35). This is a complicated issue, but is most definitely a focus of the HSR. Much more difficult for China, and currently lacking attention, will be financing several specific health issues: non-communicable disease management and control in a population that is both rapidly aging and whose lifestyle and diet (beginning in infancy with low rates of early and exclusive breastfeeding and poor quality complementary food) increase related risk; the impact of migration and urbanisation on health outcomes; periodic crises arising from the persisting and risky mix of fierce competition and lax regulation in areas like food safety and the pharmaceutical industry; and risks arising from China’s population size and density, which foments new communicable disease challenges virtually every year. In these areas, China has far fewer examples to draw on, or where solutions exist, they often cannot be reliably implemented.

It has been said that health care in China became unaffordable for the population, but that HSR might become unaffordable for the government (36). Certainly reigning in costs and improving efficiency at the same time as reducing health risks, surely an equally important component of reforming China’s health sector, presents a formidable constellation of challenges for China’s leaders.
